# Bacteremia in Patients with Sepsis in the ICU: Does It Make a Difference?

**DOI:** 10.3390/microorganisms11092357

**Published:** 2023-09-20

**Authors:** Tomáš Nejtek, Martin Müller, Michal Moravec, Miroslav Průcha, Roman Zazula

**Affiliations:** 1 Department of Epidemiology, Faculty of Military Science, University of Defence, 500 01 Hradec Králové, Czech Republic; michal.moravec@ftn.cz; 2 Department of Anesthesiology and Intensive Care, First Faculty of Medicine, Charles University and Thomayer University Hospital, 140 59 Prague, Czech Republic; martin.muller@ftn.cz (M.M.); roman.zazula@ftn.cz (R.Z.); 3 Department of Clinical Biochemistry, Hematology and Immunology, Na Homolce Hospital, 150 00 Prague, Czech Republic; miroslavprucha@gmail.com

**Keywords:** sepsis, septic shock, Sepsis-3, bacteremia, outcome, ICU, foci of infection

## Abstract

Sepsis (and septic shock) is on of the most common causes of death worldwide. Bacteremia often, but not necessarily, occurs in septic patients, but the impact of true bacteremia on a patient’s clinical characteristics and outcome remains unclear. The main aim of this study was to compare the characteristics and outcome of a well-defined cohort of 258 septic patients with and without bacteremia treated in the intensive care unit (ICU) of a tertiary center hospital in Prague, Czech Republic. As expected, more frequently, bacteremia was present in patients without previous antibiotic treatment. A higher proportion of bacteremia was observed in patients with infective endocarditis as well as catheter-related and soft tissue infections in contrast to respiratory sepsis. Multivariant analysis showed increased severity of clinical status and higher Charlson comorbidity index (CCI) as variables with significant influence on mortality. Bacteremia appears to be associated with higher mortality rates and length of ICU stay in comparison with nonbacteremic counterparts, but this difference did not reach statistical significance. The presence of bacteremia, apart from previous antibiotic treatment, may be related to the site of infection.

## 1. Introduction

Sepsis (and septic shock) remains one of the most common causes of death worldwide, including in the Czech Republic [1,2,3,4]. Considering the unprecedented demographic change with 25% of the population already aged over 60 years in Europe, further expected age increase of patients admitted to ICU, increasing length of life, antibiotic resistance, and use of immunotherapy may thus determine sepsis to become an important burden on healthcare systems in the near future [5,6,7].

Early management of sepsis, along with rapid identification of pathogens and the administration of appropriate antimicrobial therapy, has a crucial importance for clinicians to reduce mortality, improve patient outcomes, and enhance the cost-effectiveness of delivered care [8,9].

Several studies have tried to evaluate the outcome of septic patients, depending on the presence or absence of bacteremia, with inconclusive results [10,11,12,13]. Bacteremia often, but not necessarily, occurs in septic patients, and blood culture-negative sepsis is common [14]. In such a heterogenous group as critically ill patients with sepsis, the likelihood of detecting positive blood cultures varies noticeably [15]. Besides the effect of antibiotic treatment prior to blood culture sampling on culture positivity, several other factors, such as inadequate investigation technique or sample processing, patients’ age, comorbid conditions, and other characteristics were proposed as potential culprits [13,16,17,18,19]. 

Thus, although blood culture sampling is the cornerstone of sepsis diagnosing, the clinical characteristics and implications of true bacteremia in septic patients, as well as the presence of bacteremia itself, remains unclear and is still debated [18,20].

The primary aim of our study was to compare characteristics and outcome of septic patients treated at the intensive care unit (ICU) meeting Sepsis-3 criteria with and without documented bacteremia.

We evaluate the outcome of septic patients treated in the ICU according to multiple variables including demographics, severity of clinical condition, comorbidities, different sites of infection, and previous antibiotic treatment or administration.

## 2. Materials and Methods

### 2.1. Study Population

Analysis of the patient dataset from December 2012 to July 2020 obtained from the Department of Anesthesiology and Intensive Care, First Faculty of Medicine, Charles University and Thomayer University Hospital, Prague, was performed.

The following criteria were required for study inclusion: (1) Fulfilled criteria according to the Sepsis-3 definition. (2) Available results of blood cultures (BC)—at least 1 aerobic and 1 anaerobic bottle drawn at the time of sepsis diagnosis. (3) Change in clinical status less or equal to 3 h prior to admission to ICU or start of sepsis treatment.

**Figure 1:**
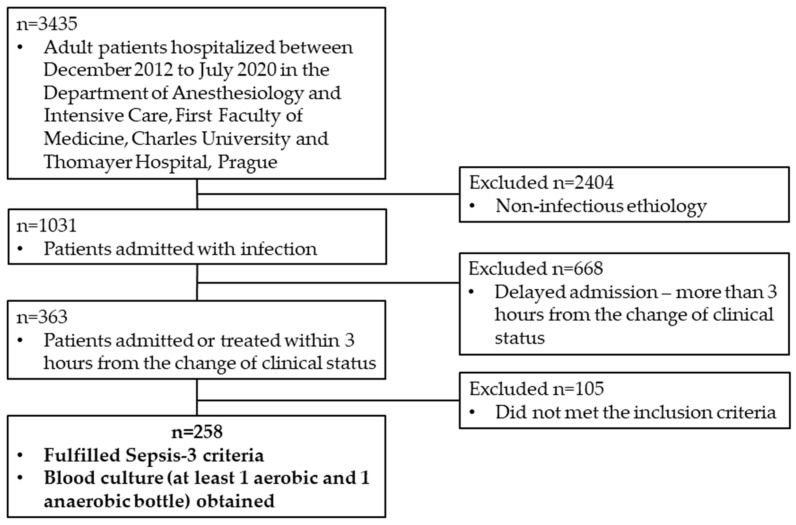
Process of patient selection and study inclusion criteria.

A total number of 258 patients met the inclusion criteria. The process of patient selection is demonstrated in Figure 1.

### 2.2. Data Collection and Laboratory Diagnostics

For each patient, the following data were collected: demographics; history and comorbidities; results of blood cultures; initial SOFA score and lactate; C-reactive protein (CRP); procalcitonin (PCT); foci of sepsis; record of previous antibiotic treatment or administration; mortality; and ICU length of stay.

Blood cultures were drawn during or before newly administered antibiotic treatment under aseptic conditions, always at least 1 aerobic and 1 anaerobic bottle (Bactec Plus—Becton Dickinson, Franklin Lakes, NJ, USA). Then, 10 mL of whole blood was inoculated per bottle and processed in the department of clinical microbiology. Each bottle was incubated in the blood culture system (Becton Dickinson BACTEC FX40—Becton Dickinson, Franklin Lakes, NJ, USA) for five days. If positive, it was cultivated by standard microbiological methods. 

Bacteremia was defined as the presence of a causative pathogen found in blood culture(s), which were thoroughly evaluated by the physician in charge and experienced clinical microbiologist according to complementary investigations, presumed or confirmed focus of infection, other culture specimens (sputum, urine, etc.), and number of positive bottles (sets). All potential contaminants were ruled out and were not evaluated further, unless recognized by additional clinical and laboratory findings as a true pathogen.

Identification of the foci of infection was determined by a combination of imaging and laboratory findings and clinical judgement of the experienced interdisciplinary team. Groups were designated as follows: (1) Respiratory; (2) Abdominal; (3) Soft Tissues; (4) Urogenital; and (5) Catheter-Related Blood Stream Infection (CRBSI) and Infective Endocarditis (IE). If more potential sources of infection were probable, they were marked as group (6) Multiple. If no conclusive source of infection was found, foci remained (7) Unknown.

Lactate, partial pressure of oxygen in arterial blood, platelet count, bilirubin, creatinine, PCT, and CRP were investigated by standard methods available bedside or in the hospital laboratory.

Patients’ histories and comorbidities were assigned to the Charlson comorbidity index (CCI) [21].

### 2.3. Statistical Analyses

Continuous data are presented as median (1st quartile—3rd quartile), and categorical data are presented as number (percentage). For comparison of continuous data, the Wilcoxon/Kruskal-Wallis test was used. For comparison of categorical data, the Chi-square test was performed. Differences in survival in bacteremic and nonbacteremic patients were analyzed using the Kaplan-Meier method. A Cox proportional hazards model was used to perform a multifactorial analysis of the influence of selected factors on survival times and *p*-value(s) ≤ 0.05 was considered statistically significant. R 4.1.2 (The R Foundation, Vienna, Austria) with extension R-Studio 2023.03.0 + 386 (Posit Software, PBC, Boston, MA, USA) was used to perform statistical analysis.

## 3. Results

Out of 258 samples, 180 (69.8%) was blood culture negative. Out of 78 (30.2%) bacteremic patients, there was 34 (43.6%) with gram-positive flora, 35 (44.9%) with gram-negative flora, 5 (6.4%) with mixed flora, and 4 (5.1%) fungi.

The representation of causative pathogen strains in blood cultures were as follows: *Acinetobacter* 1 (1.3%); *Candida* 4 (5.1%); *Enterobacter* 5 (6.4%); *Enterococcus* 4 (5.1%); *Escherichia* 8 (10.3%); *Klebsiella* 12 (15.4%); *Moraxella* 1 (1.3%); *Morganella* 1 (1.3%); *Multibacterial* 8 (10.3%); *Proteus* 1 (1.3%); *Pseudomonas* 1 (1.3%); *Sarcina* 1 (1.3%); *Serratia* 2 (2.6%); *Staphylococcus* 23 (29.5%); *Stenotrophomonas* 1 (1.3%); *Streptococcus* 5 (6.4%).

The majority of blood cultures (119; 66.1%) were drawn during ongoing antibiotic treatment. A significantly higher proportion of bacteremia was observed in the group without previous antibiotic treatment in comparison with the group with previous antibiotic administration. Bacteremia was more frequent in patients with more comorbidities.

Although initial SOFA scores did not differ between the bacteremic and nonbacteremic group, a marginally higher occurrence of septic shock was observed in the patients with presented bacteremia, but this difference in comparison with the nonbacteremic group was not statistically significant.

Monitored laboratory parameters did not statistically differ between the bacteremic and nonbacteremic group.

All selected patient characteristics, such as: sex; age; SOFA score; SOFA organ dysfunction; occurrence of septic shock; CCI and related comorbidities; record of previous antibiotic therapy; CRP; PCT; and lactate are shown in Table 1 and Table 2.

In the whole cohort, the highest percentage of bacteremia was observed in patients with CRBSI, IE, and soft tissue infection. In the subgroup of patients without previous antibiotics, CRBSI and IE (but not soft tissue infection) were the infections with the highest percentage of recorded bacteremia. Respiratory site of infection showed the lowest proportion of documented bacteremia in the whole cohort, as well as in the subgroup without previous antibiotic treatment, which was statistically significant.

A representation of the different sources of infection and occurrence of bacteremia in the bacteremic and nonbacteremic group is shown in Table 3.

**Figure 2:**
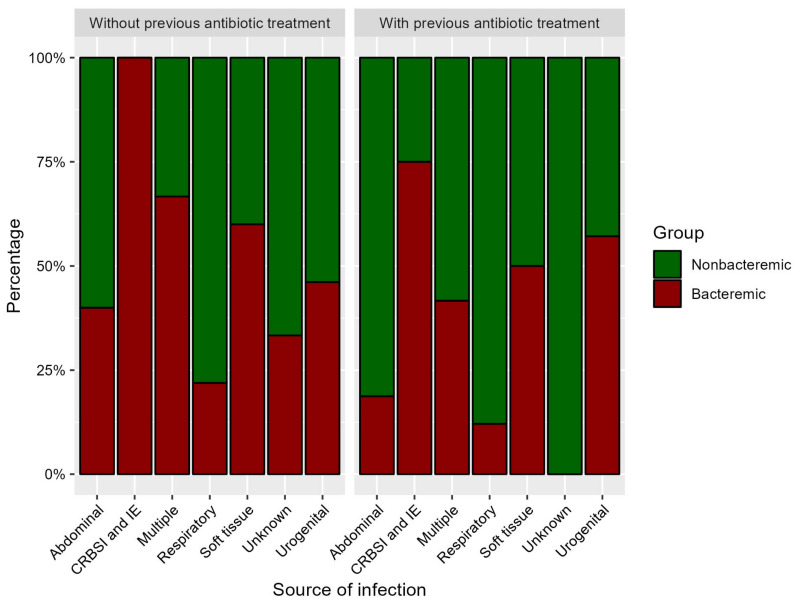
Proportion of bacteremic and nonbacteremic patients in groups with and without previous antibiotic treatment in different foci of infection.

The proportion of bacteremic and nonbacteremic patients in groups with and without previous antibiotic administration in different foci of infection is illustrated in Figure 2.

The bacteremic group, in comparison with the nonbacteremic group, showed worse survival, but this observation didn’t reach statistical significance. The length of stay in the ICU was also higher in the bacteremic group when compared to the nonbacteremic group, but the difference was not statistically significant.

**Figure 3:**
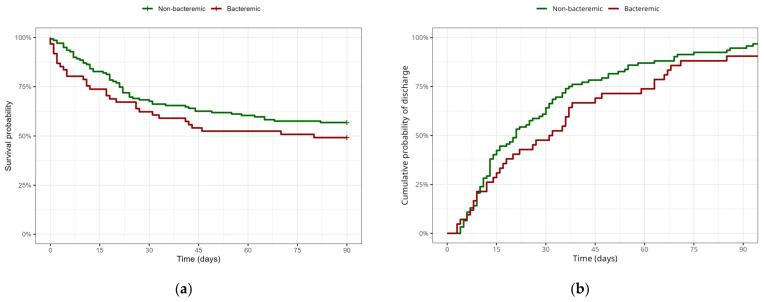
Kaplan-Meier model of survival (**a**) and ICU length of stay (**b**) in bacteremic and nonbacteremic groups.

A comparison of survival and ICU length of stay between the bacteremic and nonbacteremic groups is illustrated in Figure 3.

Better survival was observed in patients with previous antibiotic treatment, but the difference in comparison with the group without previous antibiotics was not statistically significant.

**Figure 4:**
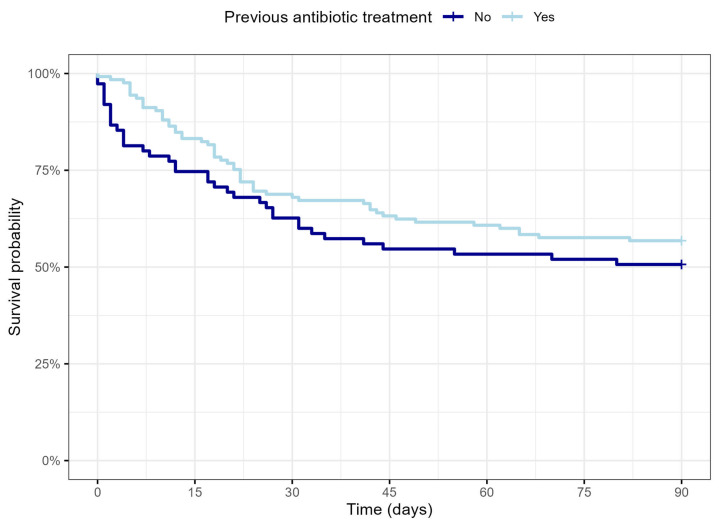
Kaplan-Meier survival analysis in patients with and without previous antibiotic treatment.

Survival analysis depending on previous antibiotic treatment is illustrated in Figure 4. 

Multivariate regression analysis showed Charlson comorbidity index variables including age, initial SOFA, and occurrence of septic shock as significantly decreasing overall survival. Neither bacteremia nor previous antibiotic treatment, as well as age, gender, or site of infection showed statistical effect on survival. 

Multiple variables and their effects on survival are shown in Table 4.

## 4. Discussion

The main observations of our study showed a very similar clinical and laboratory pattern (See Table 1) in bacteremic and nonbacteremic groups of septic patients. Nevertheless, a different outcome in septic patients with bacteremia was reported in several previously published studies [10,11,12,13,14,22]. The question of variables affecting the outcome of critically ill patients with sepsis continues to be a point of interest. As expected, multivariant analysis confirmed the severity of initial clinical condition, septic shock, and higher CCI as variables with significant effects on overall mortality in patients hospitalized in the ICU with sepsis. Apart from that, the data of our cohort showed that, although bacteremic patients had slightly higher mortality and ICU length of stay when compared to nonbacteremic patients (See Figure 3), this difference didn’t reach statistical significance. These findings are in accordance with the observations of other recently published studies performed by Kethiredy et al. (2018) and Sigakis et al. (2019) [15,18]. Similarly, a study conducted by Komori et al. (2020) with adjusted mortality according to age, sex, CCI, SOFA, occurrence of septic shock, and site of infection showed that in-hospital mortality does not differ in patients with and without presented bacteremia [20]. Based on our observations, ongoing antibiotic treatment at the time of blood culture sampling did not statistically improve survival in septic patients. Although multivariant analysis showed there is no significant influence of previous antibiotic treatment or presence of bacteremia on survival, a marginal positive survival effect of the absence of bacteremia and/or previous (any) antibiotic treatment was observed. This is supported by some previously published studies, as mentioned above. We do admit that the fundamental question whether the better outcome in patients without bacteremia is due to the absence of bacteria in the bloodstream per se vs. a more common antibiotic administration before admission cannot be fully explained, partially also by the accuracy and limitations of blood culture-based diagnosing itself [23]. Moreover, inadequate pre-ICU admission antibiotic treatment and unrecognized new onset of healthcare-associated infections could have affected these results as well, as stated in limitations [8,24,25].

However, with strict adherence to inclusion criteria, ruling in only patients admitted or treated in the ICU within 3 h after change of clinical status who have undergone blood culture sampling before newly administered antibiotics, as described in methods, and using a sufficient cohort of septic patients, we can provide relevant findings.

The incidence of culture-positive sepsis in most previously published studies range widely between 40–70%, but an incidence of only 11% was reported [15,18,26,27,28]. Bacteremia, as described in methods, presented in approximately one third of our septic patients’ cohort. Unsurprisingly, the presence of bacteremia was more frequently documented in the group without previous antibiotic treatment or administration. Relatively low occurrence of bacteremia in our study cohort may therefore be influenced by previous antibiotic treatment [29,30]. Although antibiotic treatment should not be delayed due to blood culture sampling, the drawing of blood cultures prior to antibiotics should be further encouraged [8,31].

The site of infection and its relevance to the detection of a causative pathogen in the blood is still debated. Individual host-pathogen interaction during immune response to infection, as well as the different nature of organ-specific immune system setting in the presumed origin of infection, are variables which are still not fully explained, and robust evidence for this hypothesis is lacking in the literature [17]. A higher percentage of bacteremia due to intravascular devices and multiple-source infections was reported by Jeganathan et al. (2017), whereas lung- or abdomen-related sepsis resulted in a lower proportion of recorded bacteremia [32]. Similarly, Phua et al. (2013) found that culture-positive sepsis is less common in lung-associated infections in contrast to urinary tract-, soft tissue-, and primary bloodstream-related infections [19]. Phua’s conclusion was challenged in commentary by Prost et al. (2013), who pointed to lack of information about previous antibiotic treatment [33]. In this respect, we can report a significantly higher percentage of presented bacteremia in catheter-related bloodstream infections and infective endocarditis in contrast to respiratory tract-related sepsis in patients without previous antibiotic treatment or administration.

From a national public health perspective, a surprisingly low number of studies evaluate the epidemiology, characteristics, and outcome of septic patients in the Czech Republic [34,35]. Moreover, to our best knowledge, none of them used Sepsis-3 criteria for patients hospitalized in the ICU. In this study, we described the epidemiology and outcomes associated with sepsis and septic shock defined and based on the Sepsis-3 criteria; thus, our study provides contemporary and relevant data (in a well-defined patient cohort) on current sepsis characteristics in the Czech Republic.

Our study has several limitations. First, the study was conducted retrospectively. Second, this study comes from a single-center, high-level facility, and the conclusions can be applied only for a survey population. Third, in patients admitted from other wards, sepsis diagnosis, enrolment into the study, and quality of data could have been confounded with hospital-acquired infections and previous inadequate antibiotics, which could thus affect the overall results. Fourth, there was a relatively low number of positive blood cultures, especially in the group with previous antibiotics. Finally, there was an insufficient number of patients for each studied pathogen to evaluate the microorganism-related difference in mortality. Nevertheless, the diversity of the study population, the setting, and the study design, including appropriate statistical models, may reveal different impacts of bacteremia on patients’ clinical courses and outcomes [20], which makes our study meaningful and valuable for further research in this field.

## 5. Conclusions

Bacteremic patients showed a slightly higher overall mortality and ICU length of stay in comparison to the nonbacteremic group, but this difference was not statistically significant.

The severity of the initial clinical condition, occurrence of septic shock, and higher Charlson comorbidity index, including age, are variables with significant influence on overall mortality in patients hospitalized in the ICU with sepsis.

The occurrence of bacteremia is also influenced by the site of infection.

## Figures and Tables

**Table 1:** Comparison of selected characteristics in the bacteremic and nonbacteremic group. Data are displayed as number (percentage) or median (Q1–Q3).

Demographics, Clinical and Laboratory Data		Bacteremic (*n* = 78)	Nonbacteremic (*n* = 180)	*p*-Value
Sex	Male (n)	51 (65.4%)	119 (66.1%)	1
	Female (n)	27 (34.6%)	61 (33.9%)	
Age (years)		66.5 (56.5–73)	66 (58.75–72)	0.79
SOFA (points)		9 (6–12)	9 (6–11)	0.68
SOFA organ dysfunction	Cardiovascular system (points)	3 (0–4)	3 (0–4)	
	Central nervous system (points)	1 (1–3)	1 (1–3)	
	Coagulation (points)	0 (0–1)	0 (0–1)	
	Liver (points)	0 (0–1)	0 (0–1)	
	Renal function (points)	1 (0–3)	1 (0–2)	
	Respiratory system (points)	2 (0–3)	2 (0–3)	
Septic shock (n)		30 (38.5%)	65 (36.1%)	0.83
Charlson Comorbidity Index (points)		5 (2–7)	4 (3–6)	0.69
Previous antibiotic therapy (n)		34 (43.6%)	119 (66.1%)	0.001
C-reactive protein (mg/L)		158.2 (103.7–285.6)	159.5 (82.2–251.8)	0.45
Procalcitonin (μg/L)		1.88 (0.71–15.42)	2.54 (0.75–9.78)	0.63
Lactate (mmol/L)		1.8 (1.1–3.2)	1.8 (1.2–2.8)	1

**Table 2:** Representation of individual comorbidities according to CCI. Data are displayed as number (percentage).

Comorbidity	*n* (%)
Myocardial infarction	64 (24.8%)
Diabetes with chronic complication	56 (21.7%)
Congestive heart failure	48 (18.6%)
Chronic pulmonary disease	39 (15.1%)
Cancer without metastasis	35 (13.6%)
Diabetes without chronic complication	30 (11.6%)
Leukemia	3 (1.2%)
Lymphoma	3 (1.2%)
Peptic ulcer disease	28 (10.9%)
Peripheral vascular disease	27 (10.5%)
Renal disease	26 (10.1%)
Cerebrovascular disease	21 (8.1%)
Rheumatic disease	19 (7.4%)
Mild liver disease	14 (5.4%)
Moderate or severe liver disease	12 (4.7%)
Dementia	11 (4.3%)
Metastatic solid tumor	11 (4.3%)
Hemiplegia or paraplegia	1 (0.4%)

**Table 3:** Occurrence of bacteremia in different foci of infection. Data are displayed as number (percentage).

Source of Infection	Bacteremic (*n* = 78)	Nonbacteremic (*n* = 180)	*p*-Value
Abdominal	17 (21.8%)	51 (28.3%)	<0.001
CRBSI and IE	12 (15.4%)	1 (0.6%)	
Multiple	9 (11.5%)	9 (5.0%)	
Respiratory	17 (21.8%)	90 (50.0%)	
Soft tissue	11 (14.1%)	9 (5.0%)	
Unknown	2 (2.6%)	10 (5.6%)	
Urogenital	10 (12.8%)	10 (5.6%)	

**Table 4:** Multivariant analysis of different variables on survival.

Factor	HR (95% CI)	*p*-Value
Blood culture positivity	1.4 (0.9–2.1)	0.172
Charlson Comorbidity Index	1.2 (1.1–1.3)	<0.001
Previous antibiotic treatment	1.1 (0.7–1.7)	0.639
Septic shock	1.9 (1.1–3.2)	0.013
SOFA score	1.1 (1.0–1.2)	0.045

HR—Hazard ratio; CI—Confidence interval.

## Data Availability

http://arkftn.cz/research/003/Microorganisms-Data.xlsx (Accessed on 14 September 2023).
